# Combining Integration of Care and a Population Health Approach: A Scoping Review of Redesign Strategies and Interventions, and their Impact

**DOI:** 10.5334/ijic.4197

**Published:** 2019-04-11

**Authors:** Elina Farmanova, G. Ross Baker, Deborah Cohen

**Affiliations:** 1 Institute of Health Policy, Management and Evaluation, University of Toronto, Toronto, CA; 2 School of Epidemiology, Public Health and Preventive Medicine, University of Ottawa, Ottawa, CA; 3 Canadian Population Health Initiative, Canadian Institute for Health Information, Ottawa, CA

**Keywords:** integrated care, population health, redesign interventions, determinants of health

## Abstract

**Background and aim:**

Many health systems attempt to develop integrated and population health-oriented systems of care, but knowledge of strategies and interventions to support this effort is lacking. We aimed to identify specific redesign strategies and interventions, and to present evidence of their effectiveness.

**Method:**

A modified scoping review process was carried out. Fifteen relevant examples of integrated care organizations that incorporated a broad population health approach in countries of the Organization for Economic Cooperation and Development described in 57 articles and reports were included in analysis.

**Results:**

Seven key redesign strategies and multiple redesign interventions have been identified and are described. Most commonly used redesign strategies included focusing on health and wellness, embracing intersectoral action and partnerships, addressing health in vulnerable groups, and addressing a wide range of determinants of health, including making improvements in health services. Redesign interventions included creative and innovative ways of addressing clinical and non-clinical issues such as establishing housing surgeries in primary care, establlishing vast social and provider networks to support patients with complex needs and also broadening of the scope of services, workforce redesign and other. Potential reductions in the utilization of care and costs could be derived by the wider adoption of these strategies and interventions.

**Conclusion:**

Development of integrated and population health-oriented systems of care requires the redesign of how services are organized and delivered, and how organizations and care systems operate. Combining integration of care with the population health approach can be supported by a set of cohesive strategies and interventions aimed at preventing disease, addressing social determinants of health and improving health equity at both population- and individual-level.

## Introduction

Health systems worldwide face increasing challenges from growing numbers of complex multimorbid patients, the rising costs of care and an increasing recognition of the impact that results from a failure to address the social determinants of health [1,2,3,4]. Collaborative or integrated health care delivery has proven to be effective for patients with complex medical needs [5,6,7,8] and is now seen as a necessary innovation [9] to address these challenges. Extending the benefits of integrated care to the general population, requires combining the scope of integrated care with a *population health approach* [2,10,11,12,13]. This approach to care considers a wide range of factors and interrelated conditions that influence the health of populations over the life course, identifies systematic variations in their patterns of occurrence, and applies the resulting knowledge to improve the health and well-being of those populations [11]. This approach also commonly shifts the focus to prevention, multiple determinants of health, equity in health, intersectoral action and partnerships, and understanding the needs and solutions through community outreach [14].

Neither the integration of care and nor the population health approach are novel concepts; although some general strategies and design principles have been proposed for both the integration of health services [3,15,16,17,18] and the population health approach [2,19,20,21,22,23] the linkage of these concepts remains challenging. Moreover, the concrete operationalization of these strategies is still missing in many settings. For example, it is often unclear how to expand the scope of integrated care beyond traditional health services and how to address the social determinants of health in the processes of care [24]. Some integrated healthcare organizations such as Kaiser Permanente in the U.S [25]. and Gesundes Kinzigtal in Germany [26], have successfully adapted a broad population health approach to the organization and delivery of care. These and other examples demonstrate that integrated health care systems can be successfully redesigned to incorporate the population health approach to care [2]. But such examples need to be studied systematically to inform and facilitate the broader adoption of the integrated care combined with the population health approach.

This paper presents the results of a scoping review of selected examples from countries of the Organization for Economic Cooperation and Development (OECD) of initiatives that have taken on the broad population health approach in the context of the integration of care. The review sought to answer the following key question: How can the population health approach and its elements be embedded in the context of integrated care delivery? The aim was to investigate: 1) redesign interventions that can facilitate combining integration of care with elements of the population health approach, and 2) evidence of the effectiveness of these interventions. For the purpose of this paper, we define interventions as either changes in or redesign of processes and structures in healthcare organizations or innovations with an overall objective of establishing integrated population health-based systems of care. This definition is deliberately broad so as to cover as many interventions as possible. This article also discusses real-life challenges reported in literature with regard to implementation of integrated population health-based care delivery and recommendations for its scalability.

## Methods

We modified Arksey and O’Malley’s [27] five stage scoping review process (identifying the research questions (stage one), identifying relevant studies (stage two), selecting studies (stage three), charting the data (stage four), collating, summarizing and reporting results (stage five) by adding two additional steps between stages 2 and 3. Specifically, after identifying relevant studies, we looked for the description of initiatives in these reports and searched for additional information on each identified initiative before commencing stage 4 (charting the data). We identified 27 relevant initiatives and after applying the inclusion criteria, 15 initiatives were selected for the review.

To identify relevant reports, one reviewer (EF) with the support of a librarian searched OVID-medline, Pubmed, Mendeley and EMBASE databases and screened articles based on titles and abstracts. The detailed search strategy is presented in Table 1. A hand search of bibliographies in selected articles and grey literature was also performed. Another team member (JPN) reviewed selected reports to validate their inclusion using agreed upon criteria. Inclusion criteria specified that all initial research must be drawn from the OECD countries, published between the years 2000 and 2015. In 2017, new reports were included to update the content of this review to December 2017. Relevant studies were required to describe examples of either established or new initiatives or programs for which descriptive information was available, and that were evaluated (internally or externally). Specifically, we looked for the presence of at least one interface (e.g., referral, co-location) between healthcare and non-healthcare services that addressed multiple determinants of health as evidence of the integration of medical and non-medical sectors beyond individual tiers of public health, primary care, acute care/hospitals, mental health, community care, and long-term care services. These inclusion criteria helped us identify examples of initiatives and programs designed with consideration of concepts of integrated care [15,28] and tenets of the population health approach [21]. The selected initiatives included a number of upstream interventions across sectors and levels of care that address the challenge of moving beyond the individual to the community.

**Table 1:** Search strategy.

Steps	Search terms and combinations

1	integrated care.mp. [mp=ti, ot, ab, nm, hw, kw, kf, px, rx, ui, an, tc, id, tm, tx, sh, ct, tn, dm, mf, dv]
2	population health.mp. [mp=ti, ot, ab, nm, hw, kw, kf, px, rx, ui, an, tc, id, tm, tx, sh, ct, tn, dm, mf, dv]
3	(integrated adj3 (organi?ation* or care or healthcare or hospital* or service* or policy or policies or system or systems)).ti,ab.
4	(intersectoral adj3 (organi?ation* or care or healthcare or hospital* or service* or policy or policies or system or systems or partnership or partnerships)).ti,ab.
5	Models, Organizational/
6	Delivery of Health Care/
7	Determinants of health/
8	Equity.mp. or inequity/[mp=ti, ot, ab, nm, hw, kw, kf, px, rx, ui, an, tc, id, tm, tx, sh, ct, tn, dm, mf, dv]
9	Organizations/
10	or/3–9
11	1 and 2 and 3 and 10

Search terms used:
care delivery delivery of health care determinants determinants of health equity health healthcare hospital* inequity integrated integrated care intersectoral models, models, organizational organi?ation* organizational organizations partnership partnerships policies policy population population health service* system systems

For each selected initiative we carried out an additional search of published and grey literature to collect details about interventions and results. Two team members (EF, JPN) independently reviewed each report and abstracted data using inductive reasoning and open coding to identify themes. Abstraction and analysis of data were conducted in parallel. Two team members (RB, DC) reviewed a subset of reports to validate findings and their accuracy. Disagreements associated with data abstraction and analyses were resolved in a discussion between the reviewers who abstracted data or all team members together.

We used the 7 population health elements identified by the Canadian Institute for Health Information (CIHI) (14) and the 12 determinants of health described by the Public Health Agency of Canada (PHAC) [29] to categorize population health strategies and redesign interventions, respectively. Together these population health elements and the determinants of health help identify specific foci for the population health efforts and how they have been linked to integrated care.

In assessing each initiative, we identified the level and the type of integration among medical and non-medical care and services using Leutz’s [30] continuum of integration (linkages, coordination, full integration) and the Fulop et al.’s [31] integration typologies (organizational, functional, service, clinical, normative, and systemic). Contracting arrangements that supported the level and type of integration were reviewed following the typology of Billings & de Weger [32]. Analyses of integration strategies included aspects of governance, management, funding, organization and delivery of care as proposed by Kodner and Spreeuwenberg [28]. The effectiveness of redesign interventions was assessed using results of reported evaluations for each selected initiative.

## Results

Fifteen initiatives from 9 OECD countries described in 57 articles and reports published between 2001–2017 were included in analysis. The characteristics of these initiatives are presented in Table 2. They represent a broad array of examples from short-term pilot projects, randomized controlled trials, small-scale programs in one neighbourhood or city, through to larger initiatives at regional levels. The initiatives reviewed here included both newly established care systems that adopted the population health approach from the start and established acute care systems that have developed elements of integrated population health-based systems in more recent times. Many of these initiatives were established in response to increasing rates of chronic diseases and multimorbidity thus addressed the needs of high-need/high-cost populations [9,12,33,34,35], and some have begun to combine these efforts with population health management [12,36,37].

**Table 2:** General characteristics of projects and programs (projects 1–4).

Initiative	Embrace	Liverpool City Council’s Healthy Homes Programme	New Zealand Healthy Housing Programme (also known as Counties Manukau Health)	Hennepin Health Accountable Care Organization (ACO)

**Country**	The Netherlands	UK	New Zealand	USA
**Objective**	To provide comprehensive, patient-centered, proactive, and preventive care, in addition to supporting all older adults within context of community care; To prolong ability of older adults to age in place by meeting their needs by supporting self-management, detecting changes in health status early, and preventing escalation of health- related problems.	To reduce health inequalities caused by poor quality housing conditions and improve access to health and wellbeing related services; To reduce premature deaths, primary care consultations and hospital admissions; To drive up standards in private rental sector while also addressing wider determinants of health through housing issues and other factors from access to services to lifestyles.	To improve tenant access to healthcare services in order to improve health outcomes. To reduce the risk of housing related health issues, such as an extension to the house, a transfer to a larger home, housing design improvements or creation of healthy environments, including insulation and ventilation. To identify social or welfare issues and provide a link to the appropriate social service agencies.	To treat each person holistically through the coordination of medical and social services to improve health outcomes and reduce cost; To increase use of preventive care and reduce preventable hospital admissions and emergency visits in high-risk population it serves.
**Intervention period**	2012 – present (pilot phase 2012–2013)	2009 – present	2001 – present	2011 – present
**Population size**	755 community-living adults in three municipalities	40000 properties eligible; 33000 assessments and 25000 referrals done in year one	9736 residents of 3410 homes in 2001–2007	9054
**Target population**	Older adults living in community stratified into robust, frail and complex care needs risk profiles (profiles correspond to care intensity levels)	Population living in eligible housing (neighbourhoods with high level of deprivation)	Families at high risk of infectious diseases, living in neighbourhoods with high levels of deprivation and high concentrations of public and other low-income housing.	Population is stratified based on risk and high cost; Patients with high risk/cost have highest priority for intervention.
**Sectors integrated or otherwise involved**	Primary care physicians (15 practices) and local health and community organizations (welfare service, preventive and medical care)	Public health, primary care, community-level care that includes a range of services, e.g., social care agencies, specialized care (mental health), hospitals, etc.	Joint initiative between Housing New Zealand Corporation (provider of government- funded housing) and District Health Boards that includes other tiers of care (primary care, hospitals) and social service agencies via referral	Hennepin County Human Services and Public Health Department; Hennepin County Medical Center, Level I trauma center and medium-size public hospital and safety net medical system; NorthPoint Health and Well- ness Center, and Metropolitan Health Plan
**Model of integration and/or theoretical framework**	Chronic Care Model elements (self-management, delivery system design, decision support, clinical information system), Kaiser Permanent Triangle	Initiative is rooted into councils’ understanding how quality of housing affects health and wellbeing of their residents	Socio-ecological model	Shared risk model of integrated delivery of medical, behavioral, and social services for an expanded population of Medicaid beneficiaries

**Initiative**	**Spokane and Clark counties Maternal and Child Health Inequities**	**North West London Integrated Care Pilot**	**Integrated Social Care and Health Districts in Hartberg**	**Open Care Centres for the Elderly (KAPI)**

**Country**	USA	UK	Austria	Greece
**Objective**	To reduce chronic disease in marginalized communities by improving outcomes and opportunities in early life; To transition Maternal and Child Health services from an individual-focused (mother–child dyads/family) home visiting model to a population-focused, place-based model.	To become a “beacon” for delivering integrated care; To significantly improve patient experience; To decrease emergency admissions by 30% and nursing home admissions by 10% for people with diabetes and the frail elderly through better, more proactive and coordinated care; To reduce cost of care for these groups by 24% over next 5 years.	To offer patients in need of care the possibility to stay at home; To improve and guarantee offer of health and social care in a district; To help patients and their families to find suitable care for their needs; To start and develop care programmes for relatives.	To provide older adults with primary medical, pharmaceutical and nursing care, and social and domestic assistance while they remain at their homes.
**Intervention period**	2008–2010	2013–2017	Established in 1989, the program changed and cooperation with a district hospital was added in 2000	Established in 1979, it changed throughout 1980s–90s and doubled in size in 2000s to support aging at home; (pilot phase 1979–1981)
**Population size**	NR	38000	941	17000
**Target population**	Mothers and children, pregnant women	Older adults age 75+ with diabetes	Community-dwelling older adults	Older adults age 65+, community-dwelling
**Sectors integrated or otherwise involved**	Spokane Regional Health District and Clark County Public Health led pilots and involved a great number of partners among businesses, schools, clinics.	100 general practices, 2 acute care trusts, 5 primary care trusts, 2 mental health care trusts, 3 community health trusts, 5 local authorities, and 2 voluntary sector organizations (Age UK and Diabetes UK)	Social support, preventative and primary medical services and hospital care	Social support and preventative and primary medical services
Model of integration and/or theoretical framework	Socio-ecological model Life-course approach	NR	NR	Innovative programmes aiming at socialisation of elderly, keeping them active, fit and healthy and creating awareness in their social environment.

**Initiative**	**Zorgvoorziening Zijloever (Care friendly district)**	**Integrated services for frail elders (SIPA)**	**Torbay Integrated Care Pilot**	**Gesundes Kinzigtal**

**Country**	The Netherlands	Canada	UK	Germany
**Objective**	To deliver primary medical, pharmaceutical, nursing care, social and domestic assistance to eligible persons with disabilities living in community.	To respond appropriately to needs of older persons with disabilities; To maintain and promote independence of older persons; To optimize use of community-, hospital- and institutional-based resources	To establish integrated health and social care teams within a single organization to better meet the needs of older people.	To establish more efficient and better-organized health care in cooperation with patients, health professionals and health insurers; To provide best practice health care to all patients.
**Intervention period**	Established in 1990, program undergone changes and expansion to comprehensive services in 2000s	1999–2001	2005 – present	2006 – present
**Population size**	NR	1230	145000	69000
**Target population**	Older adults age 65+ eligible on medical grounds for place in residential home.	Older adults age 64+, community-dwelling, with at least moderate disability	Older adults	All residents
**Sectors integrated or otherwise involved**	Long term care and wellfare services	Two community-based multidisciplinary teams with full clinical responsibility for delivering integrated care through provision of community health and social services and coordination of hospital and long term care.	Primary and secondary care (primary care trust that also took over hospital care and adult social care services, Torbay Council and Torbay Care Trust)	Physicians’ network and health care management company with background in medical sociology and integrated care management.
**Model of integration and/or theoretical framework**	Concept of ‘care-friendly districts’ supported by national policies.	Integrated Services for Frail Elderly delivering integrated social and health services, acute and long term care, community- based and institutional services.	“Bottom up” approach; departed from the creation of integrated health and social care team established in Brixham in 2004.	Triple Aim approach, chronic care model, innovative model of integration in its combination of logistical re-engineering of care processes, IT integration, public health and prevention measures.

**Initiative**	**Jönköping County Council**	**Kaiser Permanente (Southern California)**	**Nuka System of Care**	

**Country**	Sweden	USA	USA	
**Objective**	To improve access in the whole system of care; To deliver more care in the community; To prevent acute exacerbations of chronic disease; To increase value for patients by improving treatment, care, systems and processes	To provide high quality, affordable care to members To manage population health	To build a high-performing health system; To improve access to services; To support relationship-based model of care; To promote customer-owners’ pride and self-confidence; To honour Alaska Native culture	
**Intervention period**	1997-present	1980s-present	1998 – present	
**Population size**	340000	3.5 million	65000 plus 10000 people from remote villages	
**Target population**	Residents in geographic area stratified as: children and young people people with mental health conditions people living with drug and alcohol addiction older people	Insured members, communities and KP’s own employees	All residents in geographic area, including registered patients	
**Sectors integrated or otherwise involved**	Public health Primary care Hospital care Social care	Ambulatory, urgent and emergency care inpatient, continuing care, and virtual (for example, phone, e-mail, and Internet) settings	Local primary care Regional community hospital Tertiary care state wide hub	
**Care model and/or theoretical framework**	Chronic Care Model with a strong focus on quality improvement methods	Fully integrated health maintenance organization with a strong focus on health promotion and disease prevention	Modified Patient-Centered Medical Home	

### Population health strategies and determinants of health

As noted, each initiative was assessed for the 7 population health strategies [14] and the 12 determinants of health [29] to identify what startegies have been used and for which determinants of health. Commonly, initiatives used multiple population health strategies at the same time, including focusing on health and wellness, embracing intersectoral action and partnerships, addressing health in vulnerable groups, and addressing a wide range of determinants of health, including making improvements in health services (Table 3). Specific interventions used to implement these strategies are described in Table 4. All 15 initiatives aimed to improve population health by targeting more than one determinant of health and applying more than one intervention.

**Table 3:** Population health elements^1^ targeted by integrated population health-based care initiatives.

Population health elements	Initiatives														

	Embrace	Liverpool Healthy Homes	Healthy Housing	Hennepin Health	Maternal and Child Health	North West London ICP	Hartberg	KAPI	Zijloever	SIPA	Torbay ICP	Gesundes Kinzigtal	Nuka	Jönköping City Council	Kaiser Permanente

Focusing on health and wellness, prevention rather than illness	•	•	•	•	•		•	•			•	•	•	•	•
Addressing multiple determinants of health	•	•	•	•	•	•	•	•	•	•	•	•	•	•	•
Moving from person to populations	•^2^	•	•	•	•						•	•	•	•	•
Embracing intersectoral action and partnerships		•	•	•		•	•		•	•	•	•	•	•	•
Addressing equity/health disparities/health in vulnerable groups	•	•	•	•	•	•		•		•	•	•	•	•	
Understanding needs and solutions through community outreach		•		•								•	•	•	•
Adopting a long-term approach in care planning and delivery	•	•		•			•	•	•		•	•	•	•	•

**Table 4:** Interventions used to support population health elements and address the social determinants of health.

Population health elements	Focusing on health and wellness, prevention rather than illness	Addressing the social/multiple determinants of health	Taking a population rather than an individual orientation	Embracing intersectoral action and partnerships	Addressing equity/health disparities/health in vulnerable groups	Understanding needs and solutions through community outreach	Adopting a long-term approach in care planning and delivery

Determinants of health	Interventions						

**Income and Social Status**		Referral to welfare agencies by case manager (Embrace) Access to income support (Healthy Homes) “Navigator” or “integrator” help members connect with community-based social services (Kaiser Permanente)		Referral pathways supported by teams of referral and health, and social care co-ordinators and information technology (Torbay)	Access to healthy foods via community kitchens (Spokane, Kaiser Permanente) Help with financial deprivation by improving access to welfare agencies (Healthy Homes, Embrace)	Regular community meetings to identify needs and support referrals to welfare agencies (Embrace)	Addressing system-level barriers perpetuating health inequalities (Spokane)
**Social Support Networks**	Creating social support networks via socialisation of elderly (KAPI) Promoting social participation and preventing social isolation (Zijloevern) Social case management (social workers support patients with complex social problems) (Gesundes Kinzigtal)	Referral pathways supported by teams of referral and health, and social care co-ordinators and IT (Torbay) Discharge streamlined with systematic follow up within 72 hours and “Welcome Back Home” package from social care staff (Jönköping)	Referral of diabetic patients to neighbourhood support groups (Healthy Homes) Fostering of “working together” among networks of Alaska Native and other providers and customers-owners (Nuka) Physical exercise through social networking – KP Walks and Every Body Walk programs (Kaiser Permanente)	Red Cross, Caritas, Volkshilfe, district hospital coordinate network of social and health care providers (Hartberg)	Family Wellness Warriors Initiative – weekly support groups and gatherings for victims of abuse and neglect (Nuka)	Group meetings (life cafés) to discuss how to improve different aspects of health and well-being (Jönköping) Learning cafés to connect people with similar conditions and draw on expertise of “expert patients” (Jönköping) Referral to welfare agencies via regular community meetings (Embrace)	Fostering of ownership and governance from networks of Alaska Native providers and customers-owners (Nuka)
**Education and Literacy**	Enrolling children in early childhood education and ensuring school attendance (Healthy Homes) Family Wellness Warriors Initiative – education on prevention of abuse and neglect (Nuka) School interventions via Thriving Schools and the Fire Up Your Feet Program (Kaiser Permanente)	Referral to English language courses and employment training (Healthy Homes)			Family Wellness Warriors Initiative – workshops and training for providers and residents on abuse and neglect treatment and prevention (Nuka)	Need for education identified via regular community meetings and referral to welfare agencies is facilitated (Embrace)	
**Employment/Working Conditions**	Healthy Workplace and HealthWorks wellness programs for employees (Kaiser Permanente)	Referral to welfare agencies re: employment by case manager (Embrace) Employment training (Healthy Homes)				Need for employment identified via regular community meetings and referral to welfare agencies is facilitated (Embrace)	
**Social Environments**	Focus on socializing to help keep elderly active, fit and healthy in their social environments (KAPI) Primary care centre used as a meeting place and community hub (Nuka)	Social case management and/or referrals to welfare agencies (social workers or case managers support patients with complex social problems) (Gesundes Kinzigtal, Embrace)	Fostering of “working together” with the Alaska Native community for governance, planning and delivery of different types of care (Nuka) Physical exercise through social networking – KP Walks and Every Body Walk programs (Kaiser Permanente)	Integrated system of community-based care, offering front and second-line health and social services, incl. short- and long-term care in community and institutions (SIPA) Single point of contact in each zone (health and social care co-ordinators) (Torbay)	Family Wellness Warriors Initiative – weekly support groups and gatherings for victims of abuse and neglect (Nuka)	Regular community meetings to facilitate referral to welfare agencies (Embrace) Community engagement via locality-based advisory groups, governance, surveys, focus groups, telephone hotlines, reinforcement of “working together” attitude (Nuka)	Established regional organization, Social Care and Health District, for co-ordination and co-operation of health and social care organizations (Hartberg)
**Physical Environments**	Primary care centre used as a community hub was designed and built using traditional materials and exhibiting local arts to foster pride and confidence in local communities (Nuka)	Housing options advice, home improvements (Healthy Homes, Healthy Housing) House modifications due to disability (Healthy Housing) Re-housing due to overcrowding (Healthy Housing) Placement in stable housing (Hennepin)		Monitoring of protocols, control of budgets to allow utilization of home services, group homes, and additional services (SIPA) Co-location of health and social care teams (Torbay) Co-location and “open concept” – all providers in one open space (Nuka)	Enhancing residents’ sense of identity, security and inclusion by involving householders in home renovations (Healthy Housing)		Primary care centre used as a community hub was designed and built using traditional materials and exhibiting local arts to foster pride and confidence in local communities (Nuka)
**Personal Health Practices and Coping Skills**	Health promotion workshops (Healthy Homes, Healthy Housing) Health risks education (Healthy Housing) Safe opportunities for physical activity (Spokane, Kaiser Permanente) “Patient university” (health education and patient counselling by medical experts) (Gesundes Kinzigtal) “Healthy body weight” combining prevention with regular blood sugar level check ups (Gesundes Kinzigtal)		Physical activity integrated into all aspects of daily life, activated employees and health care professions, marketing of what matters, healthy foods being available throughout the community, and schools strengthened as the heart of health (Kaiser Permanente)	Referral pathways supported by teams of referral, health, and social care coordinators and IT (Torbay) Integrated medical care complimented by dental, behavioural, after-care, youth, elders; Family Wellness Warriors; Tribal and Traditional Services; Chiro, massage, acupuncture (Nuka) Integrated approach with physical activity integrated into all aspects of daily life, activated employees and health care professions, marketing of what matters, healthy foods being available in the community, and schools strengthened as the heart of health (Kaiser Permanente)	Self-management support and prevention for frail and complex care needs via community meetings and case manager (Embrace, Nuka) Health and fitness promotion for elderly with focus on socialisation (KAPI) Traditional Healing Clinic for acute or chronic pain, behavioural health and counselling (Nuka) Family Wellness Warriors Initiative – fostering individual/community skills to cope and respond to abuse and neglect and its prevention (Nuka) Increasing access to healthy foods via community kitchens (Spokane, Kaiser Permanente)		Patients (customer-owners) and providers are encouraged to use phone, email and text for routine monitoring and some preventative screening (Nuka) Patient-facing online tools for managing preventive and chronic care to increase patient agency in health promotion (Kaiser Permanente) Patient self-management and shared decision-making (Gesundes Kinzigtal, Nuka, Kaiser Permanente, Jönköping)
**Healthy Child Development**	Child immunisations (Healthy Housing, Nuka) Reducing child abuse and neglect (play and learn groups) (Spokane)			Pre- and post-natal care, including advising on birthing options and carrying out six-weekly check-ups as part of primary care services (Nuka)	Increasing access to first trimester prenatal care for marginalized women (Spokane)		
**Biology and Genetic Endowment**				No interventions noted			
**Health Services**	Individual treatment plans and goal-setting agreements between doctor and patient (Gesundes Kinzigtal, Nuka, Kaiser Permanente) Follow-up care and case management (Gesundes Kinzigtal, Nuka, Kaiser Permanente) System-wide electronic patient records (Gesundes Kinzigtal, Nuka, Kaiser Permanente) Intervention programme for patients with chronic heart failure (Gesundes Kinzigtal) ‘Healthy body weight’ combining prevention with regular blood sugar level check ups (Gesundes Kinzigtal, Kaiser Permanente) Referrals to paediatric and women’s clinics with coordination from primary care team (Nuka)	Healthy Homes on Prescription in primary care (Healthy Homes) Healthy housing surgeries in primary care (Healthy Homes) Multidisciplinary team working with all family members to enable behavioural change (Nuka) General practitioner and nurse-led case management aim to address the emotional, psychological and social determinants of health (Nuka) Health and self-management education for patients and families/caregivers (Jönköping, Kaiser Permanente) Supplementing medical records with data from the corrections department, foster care system, housing providers, and other local agencies to identify those whose health may be at risk because of nonmedical issues (Hennepin Health)	Stratification into (A) no complex care needs and with low frailty level; (B) frail at risk of complex care needs; and (C) complex care needs (Embrace) Stratification into 1) children and young people, 2) people with mental health conditions, 3) people with drug and alcohol addiction, 4) older people (Jönköping) Population risk stratification based on Kaiser’s Know your population model (Torbay) or simple assessment scales (NWL; Hennepin) Patient panel from registered list of patients (Nuka, Kaiser Permanente) “Esther model” and “Mrs Smith” provided basis for designing care pathways for all older people (Jönköping, Torbay)	Informal/formal multidisciplinary teams coordinate & provide care (Torbay, Embrace) Community-based multidisciplinary teams responsible for health and social services and coordinating hospital and nursing home care (SIPA, Hartberg) Inter- and multi-disciplinary group meetings to develop unified view of patients’ care and system navigation (NWL, Embrace, Nuka, Kaiser Permanente, Jönköping) Case managers for medical and social issues, liaising with physicians, and following patients throughout care trajectory, assuring continuity and easing transitions between hospital and community (SIPA, Nuka) Integrated system of community-based care, offering front and second-line health and social services, incl. short- and long-term care in community (SIPA) Whole-system approach, with hospitals, primary health care and other community services in partnership with zones (Torbay) Mental health services integrated using a ‘hub and spoke’ model (Torbay) Integrated medical services (primary care, women’s health, paediatrics, optometry, urgent care) complimented by dental, behavioural health, family wellness, tribal and traditional services, chiro, massage, acupuncture “The Esther model” –network of health and social care organizations; redesign of intake and care transfer process across the continuum of care; team-based telephone consultation (Jönköping)	Increasing access to first trimester prenatal care for marginalized women (Spokane) “Esther model” provided basis for designing care pathways for all older people (Jönköping) Monitoring of interdisciplinary protocols (nutrition, falls, congestive heart failure, dementia, depression, medication, vaccination) (SIPA)	Outreach conducted centrally at region and at medical centers via telephone, secure messaging, mail (Kaiser Permanente) Social worker goes on rounds with a local nonprofit’s street outreach team to find homeless members (Hennepin Health)	Established regional organization, Social Care and Health District, for co-ordination and co-operation of health and social care organizations (Hartberg) Established primary care trust to commission community health and social care services (Torbay) Developed integrated management structure for primary care trust and adult social services (Torbay) Established single management system (Torbay) Established IT tool to bring together medical and social care records from different provider organizations in one location (NWL) Making and sustaining large-scale changes to create an integrated care system capable of improvement (Jönköping) Complete care with panel management and regional safety nets to identify and respond to the needs of patients (Kaiser Permanente)
**Gender**	No gender-specific interventions noted with the exception of interventions for marginalized expectant mothers as part of the Spokane and Clark counties Maternal and Child Health Inequities initiative						
**Culture**					Providers acknowledge and incorporate importance of culture to well-being (Healthy Housing) Engagement of tribal elders and the Alaska Native community in design and delivery of care (Nuka)		Traditional healing provided by tribal doctors whose skills are culturally relevant (Nuka) Governance from Alaska Native community (Nuka)

#### Focusing on health and wellness

Health promotion was embedded in practice-level interventions and also in broader system changes that promoted wellness-oriented health and social care services. At the practice level, many initiatives focused on traditional behavioural modification approaches for high-risk groups as a key strategy for preventing diseases. At the broad system level, creation of new supportive environments to promote better personal and population health practices for broader populations [36,38,39] was observed. For example, Kaiser Permanente’s Total Health program [40] employed health promotion as a philosophy of care and used a multipronged strategy to address healthy eating and active living by fostering strong supportive environments across local communities.

Relationships between clinical staff and community partners were used widely to create supportive programs and environments addressing the needs of local populations (e.g., early childhood education opportunities [41], violence prevention programs [36], safe opportunities for physical activity [46], community kitchens [41] and farmer’s markets [42]). In these programs, health promotion and prevention efforts were often blended with traditional self-management supports [9,12,43,44]. A major focus centered on physical activity and nutrition, and attention to other determinants of health was less visible.

#### Embracing intersectoral action and partnerships

A variety of ways were used to organize intersectoral work. At an organizational level, many initiatives created an alliance of partners, defined as a strategic collaboration and cooperation among the parties who deliver services [32]. In some cases, organizations developed an elaborate system of committees and other structures to reinforce intersectoral action. At the care delivery level, multidisciplinary teams were used commonly to address the clinical and non-clinical needs of their patients. Application and monitoring of interdisciplinary protocols was used frequently in organizing and coordinating care along with more informal exchanges among the providers from different sectors. In the majority of initiatives reviewed, health sector organizations played the lead role in planning and implementing the intersectoral work. However, when the focus was on the broader determinants of health (e.g., housing), the social sector tended to take leadership. For example, in New Zealand, the Healthy Housing Program created a successful partnership between the housing and the health sectors [45]. A culture of partnership and collaboration was present from the beginning in this program and facilitated its success.

#### Addressing health in vulnerable groups

Care for vulnerable populations (e.g., frail older adults with complex needs, individuals with mental health and addictions issues, and others with limited access to health care and services) was delivered by multispecialty medical and multidisciplinary team-based care with intensive disease and case management, and an emphasis on prevention and self-management. Both professional care networks and informal (social) networks were used to provide support to patients and families whose complex needs impacted their health. The “Esther Network” in Jönköping, Sweden, for example, is a network of caregivers, clinicians, patients, and families working together to improve care for patients with complex needs who require coordination of hospital, primary, home, and community care [46].

#### Redesigning and building capacity to address the determinants of health

Health services redesign received significant attention in all reviewed initiatives. Adoption of the population health approach in health care requires system redesign to develop capacity to carry out both population-based and individual-level initiatives aimed at preventing disease, addressing social determinants of health and improving health equity [4]. In our sample of initiatives, this redesign effort commonly included the development of comprehensive, coordinated systems of community-based care, offering public health services, primary and secondary health, and social services [9,39,41,47,48,49,50,51,52] and also redesign of the delivery system to support these services [9,36,40,47,50,53,54]. For many organizations such transformations required substantial reconfiguration of services, including investments in home and community care, and strengthening of primary care [9,12,33,35,36,44,55]. Redesigning care delivery often included efforts to segment and stratify populations based on risk/needs profiles. These profiles were formulated based on complexity of medical conditions [33,34,54], diseases and population demographics [33,34,56], costs 25, 27] or their combination [25,50,53,57]. The types of care offered to population segments were transformed and included elements such as mechanisms for the coordination of care [9,33,50,51,54,58], referrals [36,45,48,54,59], case management [33,34,36,43,53], follow-ups [36,43,60,61] and team-based care [33,34,36,43,50,50] to help address specific needs of the population segments.

All initiatives reviewed featured the use of interdisciplinary or multidisciplinary teams as a substantive component of the interventions. We observed that efforts to address the social determinants of health were supported by two specific health human resources strategies: 1) introduction of new roles to complement membership on traditional interdisciplinary or multidisciplinary teams [50,62], and/or 2) expansion of mandates for existing roles (e.g., nurse care managers) [36]. New roles included care coordinators, housing or social service navigators [43,63], healthy home advocates [48], health visitors and family assistants [39], health care managers and multidisciplinary group coordinators [9,50]. In addition to new and expanded roles, some initiatives introduced protocols for delegation of care which allowed team members to practice at the top of their scope of practice. [36,43].

Taken together, the experience of these initiatives points to the importance of building and using social capital effectively [64] as a strategy to improve population health outcomes. Socializing clients with other clients was used both as prevention and rehabilitation strategy in elder care to prevent social isolation and overutilization of services [39,52], and to leverage health promotion efforts in school-aged children and adults [40]. Networking among different care providers from different (health and non-health) sectors was used often to facilitate social networking among patients and their families. In Hartberg, Austria, for example, a network of several organizations, including religious and political groups, and a district hospital coordinated the provision of health and social care as one care package [51]. The blending of health and social services supported viable social connections in this community and resulted in improved quality of care [51]. In another example, medical facilities of the Nuka System of Care in Alaska were used as a meeting place and a community hub to foster relationships and to strengthen social capital in the community [36].

### Integration and redesign of care systems

Combining integration of care with the population health approach requires changes to the way services are organized and delivered, and to the way organizations and care systems operate. As part of the analyses, we examined the degree and the type of integration [30] and the contracting models [32] used to support integration of care and embedded population health elements (**Box 1**). Details of these analyses are presented in Table 5. A range of levels of integration from linkages between agencies to full integration was observed in this sample of initiatives, but co-ordination, which refers to structures or processes created to facilitate provision of care across separately functioning sectors [30], was used most commonly. A range of co-ordination strategies from simple forms of communication and networking between providers through to more structurally-based arrangements were identified and are consistent with literature on this topic [65].

**Box 1: Definitions of the levels and types of integrated care, and types of contracting models.**

Levels of integration:^3^

- Linkage refers to lose organisational ties (linkages) among teams and organisations that form an informal network of providers either within a single-care or across the continuum of care between community and hospital or specialist services. Service delivery is supported by referrals and provision of information.
- Coordination describes explicit infrastructure installed to coordinate care across acute and other systems. Coordination is a more structured form of integration than linkage, but it operates through the separate structures of current systems. Coordination focuses on persons receiving services simultaneously or sequentially from two or more systems of care on either a short- or a long-term basis.
- Full integration creates new programs or units where resources from multiple systems are pooled. Fully integrated programs gain control of resources to define new benefits and services that they control.

Typologies of integrated care:^4^

- Organisational integration refers to structures that bring organisations together, for example, by mergers and/or structural change or virtually through contracts between separate organisations
- Functional integration describes how non-clinical support and back-office functions are integrated, such as by using electronic patient records.
- Service integration describes how different services provided are integrated at an organisational level, such as through multidisciplinary teams.
- Clinical integration refers to medical services integrated into a single or coherent process within and/or across professions, such as through use of shared guidelines and protocols.
- Normative integration refers to shared values and commitment to coordinating work that enable trust and collaboration in delivering healthcare.
- Systemic integration describes the coherence of rules and policies at all organisational levels.

Types of contracting models:^5^

- Alliance Contracting Model – a contract between the owner, financier, or commissioner and an alliance of parties who deliver the project or service.
- Lead provider/Prime contractor model – a model where one provider is given the responsibility through a contract for subcontracting to other providers for the various aspects of care to both deliver care and also to ensure all different aspects of care are fully integrated, bringing together the previously episodic providers of care into a single pathway.
- Accountable Care Organisations – groups of health care providers from primary and secondary care levels who work together to coordinate and streamline clinical care and a range of non-clinical interventions at an individual and population level in a cost-effective way.
- Outcome-based Contracting and Commissioning – is a “performance-based” contracting focusing on results rather than activities, defining clear performance expectations and measures, providing incentives and monitoring performance.

**Table 5:** Level and type of integration, contracting and partnership arrangements.

Initiative, Country	Level of integration	Type of integration	Contracting model

Torbay, UK	Full	Organizational Functional Service Clinical System	Alliance Contracting Model Contract between Torbay Care Trust (now Torbay and Southern Devon Health and Care NHS Trust) and alliance of parties who deliver integrated care and services. All parties share risk and collectively own opportunities and responsibilities to deliver care and services
SIPA, Canada	Full	Organizational Service Clinical	Lead provider/Prime contractor model Two community-based multidisciplinary teams in two areas in Montreal took full clinical responsibility for delivering integrated care through provision of community health and social services and coordination of hospital and long term care
Nuka, USA	Full	Organizational Service Clinical Normative System	Alliance Contracting Model Co-ownership and co-management agreement between Southcentral Foundation and the Alaska Native Tribal Health Consortium (ANTHC); Provision of care is based on integration among the providers and types of care/services and inter-provider and -agency referrals. Financial risk and benefits from efficiencies, better coordination or better population management are shared across the system.
NW London ICP, UK	Coordination	Organizational Functional Service Clinical	Alliance Contracting Model Alliance partnership of commissioners (clinical groups and local authorities), voluntary sector and providers from social care and other sectors governed b the Integrated Management Board. Funding surplus from efficiencies is shared across the partnership and a flexible resource envelope is made available to each multidisciplinary group for care planning, case conferences, and performance reviews.
Embrace, the Netherlands	Coordination	Organizational Service	Alliance Contracting Model University of Groningen in partnership with health insurance company Menzis and health care organization Meander initiated Embrace (public-private partnership); Formal cooperation agreement was struck between community organisations (welfare organizations and municipalities) and health care organisations (general practitioner practices), home health care organisations, homes for the elderly, nursing homes and hospitals, and elderly associations to leverage the unique skills and expertise of each organization
Healthy Housing, NZ	Coordination	Organizational Service	Alliance Contracting Model Joint initiative between Housing New Zealand Corporation (provider of government- funded housing) and District Health Boards that includes other tiers of care (primary care, hospitals) and social service agencies Alliance agreements are used to share responsibility between partners and Healthy Housing program
Hennepin, USA	Coordination	Organizational Functional Service Clinical	Accountable Care Organisations Four partner affiliates of the Hennepin County government (Hennepin County Medical Center, North-Point Health and Wellness Center, Hennepin County Human Services and Public Health Department) created Hennepin Health ACO by signing a business agreement to share full financial risk for newly enrolled Medicaid beneficiaries.
Hartberg, Austria	Coordination	Organizational Service Clinical Normative	Alliance Contracting Model Initially established as a network of social care and health organizations, including Red Cross, Caritas and Volkshilfe; district hospital added later to assure a full continuum of care for patients within the network. Principal task is to coordinate the network between the different social and health care providers in a district.
KAPI, Greece	Coordination	Organizational Service Clinical	Alliance Contracting Model Ministry of Health and Welfare and Association of Volunteer Workers set up a pilot KAPI; Since their institutionalisation, state transferred the responsibility of their management and operation to local municipalities and KAPI have been operating with the contribution of volunteer organisations, the Greek Red Cross and the Christian Youth Organisation. There is a cross-country network of KAPI, each KAPI is a legal entity with a board of directors and a network of local partners to support provision of health care and social assistance. KAPI are also part of the National Social Care System cooperating with Day Protection Centres for the Aged and Health Units.
Zijloever, ND	Coordination	Organizational Service Clinical	Alliance Contracting Model Based on co-operation between health care (general practitioners and hospitals), care (home care, institutions for psychiatric patients, the handicapped and the elderly) and prevention. Operates as care-friendly district characterized by the provision of residential care outside an institution, small-scale community-based care and services, co-location of all providers, and incorporation of welfare facilities.
Gesundes Kinzigtal, Germany	Coordination	Organizational Functional Service Clinical Normative System	Outcome-based Contracting and Commissioning A joint venture between a network of physicians in Kinzigtal and a care management company, OptiMedis AG; An outcome-based contract is set up and through this contract clinical and financial incentives are aligned in the management of integrated care; Holds contracts with statutory health insurers (“sickness funds”) to integrate health and care services for their insured populations, covering all age groups and care settings. Collaborates with community groups including gyms, sports clubs, education centres, self-help groups and local government agencies.
Jönköping County Council, Sweden	Coordination	Organizational Service Clinical Normative System	Alliance Contracting Model Governed by the county council, Esther organizing committee and small networks of Esther coaches in each municipality Provision of care is based on integration among the providers and types of care/services and inter-provider and -agency referrals. Cost savings are reinvested across the continuum of care.
Kaiser Permanente (Southern California)	Coordination	Organizational Functional Service Clinical Normative	Alliance Contracting Model Formal contractual agreements among Kaiser Foundation Health Plan (insurance company), Kaiser Foundation Hospitals (KFH), and Southern California Permanente Medical Group.
Maternal and Child Health, USA	Linkage	Organizational Service Clinical	Alliance Contracting Model Two local health departments, Spokane Regional Health District and Clark County Public Health initiated transition to place-based integrated care and maintained funding. Stakeholders included local boards of health, school partners, advisory boards, and non-profit agency leaders.
Healthy Homes, UK	Linkage	Organizational Service	Alliance Contracting Model Alliance between Liverpool City Council and Partners in public health, primary care, community-level care that includes a range of services, e.g., social care agencies, specialized care (mental health), hospitals, etc.; Provision of care is based on inert-agency referrals.

Most initiatives addressed several types of integration [66], including organizational, functional, service, clinical/medical, normative, and system integration (Table 5). The level and type of integration were supported by a variety of contracting arrangements and partnership(s) (Table 5). Alliances were used in more than half of reviewed initiatives to co-ordinate complex care between service sectors, settings, types of organisations, types of care and between different providers.

Given the diversity of partners, and the need for collaboration, governance arrangements were crucial and appeared to be largerly determined by local circumstances. Many initiatives noted the important role of an “integrator” in the governance structures [67,68] and a collaborative approach that allows for joint action and consideration of interests and concerns of all parties.

A mix of public, private and grant funding was commonly used to support transformation to an integrated population health-based system of care. To overcome difficulties with separately funded sectors, types of care, settings and professions, many initiatives attempted to align and “pool” funding by using a mixture of per capita funding [25,36,50,63,69], service reimbursement [44,50] and payment-for-performance incentives [44,50]. Several initiatives used innovative shared-savings contracts and incentives that provided flexibility, rewards for efficiency and improved quality, and also allowed reinvesting savings into less expensive primary and community-based care [50,63,68].

### Effectiveness of interventions supporting integration of care and population health

Combining integration of care with the population health approach requires multifaceted interventions (i.e., an intervention with two or more components). The type and the level of evidence on the effectiveness of these interventions are limited and uneven across this sample of initiatives. Evaluations concentrated on the impact of integrated care on utilization outcomes such as admissions to hospitals, visits to emergency rooms, hospital length of stay and outpatient visits [9,12,33,46,48,53,63,70]. The majority of initiatives that reported before and after comparisons of utilization impacts of the interventions reported statistically significant benefits for the access to care and services, including same day appointments [34,41,49,70,71]; reductions in wait time for referrals [35,70]; reductions in emergency room use, hospital admissions and 30-day readmissions, and length of stay [9,47,48,53,57]; increased uptake of screening and immunizations [47,48,61]; reductions in overall morbidity and mortality rates [47,53,59], and overall quality of care [9,51]. Two randomized controlled trials (RCT) were included [12,33]. The Embrace RCT measured and reported a higher level of perceived quality of care in the intervention than in the control group [12]. The SIPA RCT measured differences in utilization and costs of care between the intervention and control groups, and reported no significant differences in utilization and costs of emergency room, hospital acute care, and nursing home stays between the groups [33]. With regard to costs, some initiatives demonstrated the potential for cost-containment [47,56,72], cost-savings [12,40] and reduction of cost of care [44].

Some initiatives reported descriptive evaluations of organizational changes and outcomes [36,41,50,54,57], but few granular details were provided. Reported changes included increased perceived level of implementation of integrated care [12], improved processes and pathways of care [41,50,51], established coordination of care [50,57], efficient delegation of services [36,43], and reduction in wait time from identification of needs to referrals [9].

Few initiatives monitored population health indicators [73], but improvements were noted for the quality of housing [45,48,59], feelings of safety, security and trust [35,54,58,72], lifestyle changes (e.g., healthy eating, physical activity) [40,69], secure and favourable conditions in early childhood [41], expansion of social networks [39,47,51], and public engagement [72]. Interview data was used commonly to identify these improvements. In addition, instruments such as indices of deprivation [48,74] and patient-reported outcomes measures (PROMs) [58] were reported.

## Discussion

The purpose of this scoping review was to identify and report on redesign strategies and interventions that facilitate development of integrated and population health -oriented systems of care. Clearly, combining integration of care with the population health approach requires some level of integration of health and non-health services (and resources) to coordinate actions among healthcare organizations, public health agencies, social and community organizations. It also requires re-orientation of healthcare services, and implies an expansion of organizational mandates and a more comprehensive packaging of medical and non-medical services for all population groups [75]. Several strategies supported such re-orientation efforts, including focusing on health and wellness (including improvements in personal health practices and coping skills), embracing intersectoral action and partnerships, addressing health in vulnerable groups, and addressing health and social needs in populations. Each of these strategies included a set of interventions to address both clinical and non-clinical issues to complement health services (Table 4). Many interventions included innovations with comprehensive approaches to care, such as using navigators for community-based services [58], establishing housing surgeries in primary care [48], combining physcial activities with social networking [40,76], using clinical facilties to facilitate social connections in a community [72], establlishing vast social and provider networks to support patients with complex needs [35,61], innovative patient education approaches [37], and other. Addressing non-clinical issues is an emerging area of practice in integrated care that requires broadening the scope of interventions and the services provided in order to influence health of communities [77]. A continuum of strategies from health promotion [36,37,38,40,45,58,72,76] to intersectoral interventions that can support broad populations in a community [36,43,47,69], promote nurturing relationships [9,50,54,72,78] and empower vulnerable groups [9,12,44,49,57,72,79] is necessary.

Efforts to embed the population health approach within integrated care face a number of challenges. Initiatives reviewed here reported difficulties with population segmentation and the planning of needs-based services [33,34,39], difficulties with funding mechanisms and models of care that can accommodate the interactions of multidisciplinary teams [33,57], challenges with hierarchical management, governance and accountability of integrated care systems [9,53], and with adapting traditional evaluation strategies to assess the complexities of integrated population health-based type of care [9,41,44]. The crucial role of the “integrator” as well as the need for creativity and innovation with the use of funding and programming [39,41,60,68,71,80] are often key to shifting traditional care to integrated and population health-based systems of care. Although evidence on the effectiveness of multifaceted redesign interventions on the integration of care or the population health approach is limited, some of the reviewed initiatives have demonstrated that integration of health and non-health sectors can provide benefits in the utilization of care [9,47,48,53,57] and costs [9,36,48]. There is a need for expanded assessments to evaluate the clients’ needs for both social and medical care to better understand the effectiveness of multifaceted intervetnions [33,81]. Few measures of population health were reported although some reports suggested improvement in outcomes. Integrated, population focused care may benefit from more flexible teamwork to address care challenges, and from broader sharing of knowledge and experiences [36,43,50,69], as well as better metrics and innovative evaluation approaches [2,82,83]. More broadly, there is a need for continued learning about the selection of implementation strategies in different contexts, both at program and system level to facilitate transition toward integrated population health-based systems of care [2,4,19].

Several of the initiatives reviewed here, including the Nuka System of Care, Jönköping County Council, Kaiser Permanente’s programs and Gesundes Kinzigtal, have been spread and scaled up elsewhere. These efforts have had varied degrees of success due to a combination of political, economic, societal, cultural and organizational factors [35,55,68,69,78]. These experiences underline the challenges of replicating entire integrated care systems; but they also suggest that specific strategies, techniques, and innovations may be transferable to other sectors, settings, types of organizations, types of care and multidisciplinary groups [55,69]. For example, the introduction of features of the Nuka system into three primary care practices in Scotland has had positive effects in improving access to care, and patient and staff experience [55]. But establishing Nuka-style systems in Scotland required careful planning and nimbleness to adapt this model to local settings and context [55]. Developers of Gesundes Kinzigtal argue that models, interventions and evaluation frameworks that are rooted in the scientific literature and have shown to be effective are widely replicable despite context-specific features [68]. But successful replication depends on a number of conditions [68]. First, these efforts require a shared vision to go beyond traditional institutional boundaries in the planning of health interventions that address improving population health [68]. Second, a local organization needs to plan and deliver interventions while involving and communicating with key stakeholders [68]. Third, the population served needs to be defined and perhaps limited in size to enable effective use of resources and to facilitate networking among providers who develop local solutions to issues. Fourth, an innovative culture and fostering of collaborative efforts is required to derive value from new relationships for all stakeholders [68]. Such prerequisites may assist in the spread and scale up of programs that integrate population health and social care interventions with health services to address the increasing burdens of multi-morbidity and the determinants of health.

## Limitations

There are several limitations to this study. As a scoping review this study is not a comprehensive synthesis of the literature to cover the entire field of population health and integrated care. Data was limited to information included in published reports. There are likely other examples of integrated population health-based systems of care that have not yet been reported in the literature. Future studies might augment published data with interviews with studies’ authors to validate interpretations and extend the findings.

## Conclusions

We reviewed evidence from 15 integrated and population health-based initiatives from 9 OECD countries and identified specific implementation interventions and strategies, and their impact. This review suggests that improving population health requires co-ordinated efforts of many organizations, and new program elements and partnerships that extend beyond integration of health services. Combining integration of care with the population health approach requires a set of cohesive strategies to design comprehensive medical and non-medical care for defined population groups, and to redesign service organization and delivery. Further study is needed on how population health thinking and efforts support integrated care and vice versa, and the need for continued learning about redesign interventions at a program and a system level.

## Footnotes

Based on published recommendations for key determinants of health from the Public Health Agency of Canada: https://www.canada.ca/en/public-health/services/health-promotion/population-health/what-determines-health.html#key_determinants.

Targets general population of older adults.

The level of integration is defined according to Leutz W. Five Laws for Integrating Medical and Social Services: Lessons from the United States and the United Kingdom. *Milbank Q*. 1999; 77(1): 77–110.

The typologies of integration are described according to Fulop N, Mowlem A, Edwards N. Building integrated care: Lessons from the UK and Elsewhere. *NHS Confed London*. 2005: 3–15.

The models are consistent with Billings J, de Weger E. Contracting for integrated health and social care: a critical review of four models. *J Integr Care*. 2015; 23(3): 153–175. DOI: https://doi.org/10.1108/JICA-03-2015-0015.

## References

[1] Contandriopoulos, A-P, Denis, J-L, Touati, N and Rodriguez, C. The integration of health care: Dimensions and implementation. Work Pap N04-01 2003 Groupe Rech Interdiscip en sante, 2003; 31.

[2] Alderwick, H, Ham, C and Buch, D. Population health systems Going beyond integrated care, 2015; 2 London, UK: The King’s Fund Available from: https://www.kingsfund.org.uk/sites/default/files/field/field_publication_file/population-health-systems-kingsfund-feb15.pdf.

[3] Stein, KV, et al. Perspective paper Towards people-centred health services delivery: A Framework for Action for the World Health Organisation (WHO) European Region. Int J Integr Care [Internet], 2013; 13(12): 5–7. Available from: http://www.ijic.org/index.php/ijic/article/view/1514/2358 DOI: 10.5334/ijic.1514 PMC3886596 24409110

[4] Neudorf, C. Integrating a population health approach into healthcare service delivery and decision making. Heal Manag Forum, 2012; 25(3): 155–9. DOI: 10.1016/j.hcmf.2012.07.008 23252332

[5] Ivbijaro, GO, Enum, Y, Khan, AA, Lam, SS-K and Gabzdyl, A. Collaborative Care: Models for Treatment of Patients with Complex Medical-Psychiatric Conditions. Curr Psychiatry Rep [Internet], 2014; 16(11): 506 Available from: http://link.springer.com/10.1007/s11920-014-0506-4 DOI: 10.1007/s11920-014-0506-4 25218604 PMC4163191

[6] Allen, D, Gillen, E and Rixson, L. The Effectiveness of Integrated Care Pathways for Adults and Children in Health Care Settings: A Systematic Review. JBI Libr Syst Rev, 2009; 7(3): 80–129. DOI: 10.11124/01938924-200907030-00001 27820426

[7] Ouwens, M, Wollersheim, H, Hermens, R, Hulscher, M and Grol, R. Integrated care programmes for chronically ill patients: a review of systematic reviews. Int J Qual Heal care J Int Soc Qual Heal Care/ISQua [Internet], 2005; 17(2): 141–6. Available from: http://www.ncbi.nlm.nih.gov/pubmed/15665066 DOI: 10.1093/intqhc/mzi016 15665066

[8] Mitchell, GK, Burridge, L, Zhang, J, Donald, M, Scott, IA, Dart, J, et al. Systematic review of integrated models of health care delivered at the primary-secondary interface: How effective is it and what determines effectiveness? Australian Journal of Primary Health, 2015; 21: 391–408. DOI: 10.1071/PY14172 26329878

[9] Thistlethwaite, P. Integrating health and social care in Torbay Improving care for King’s Fund, 2011; 3.

[10] Kindig, DA. Understanding population health terminology. Milbank Quarterly, 2007; 85: 139–61. DOI: 10.1111/j.1468-0009.2007.00479.x 17319809 PMC2690307

[11] Kindig, D and Stoddart, G. What is population health? Am J Public Health [Internet], 2003; 93(3): 380–3. Available from: http://www.ncbi.nlm.nih.gov/pubmed/12604476 DOI: 10.2105/AJPH.93.3.380 12604476 PMC1447747

[12] Uittenbroek, RJ, Kremer, HPH, Spoorenberg, SLW, Reijneveld, SA and Wynia, K. Integrated Care for Older Adults Improves Perceived Quality of Care: Results of a Randomized Controlled Trial of Embrace. J Gen Intern Med [Internet], 2017; 32(5): 516–23. Available from: http://link.springer.com/10.1007/s11606-016-3742-y DOI: 10.1007/s11606-016-3742-y 27271728 PMC5400746

[13] Petch, A. Integration of Health and Social Care, 2016; 3 Available from: http://www.gov.scot/Topics/Health/Policy/Adult-Health-SocialCare-Integration.

[14] Huynh, TM. Population Health and Health Care, Exploring a Population Health Approach in Health System Planning and Decision-Making Ottawa, ON; 2014.

[15] Kodner, D. All Together Now: A Conceptual Exploration of Integrated Care. Healthc Q [Internet], 2009; 13(sp): 6–15. Available from: http://www.longwoods.com/content/21091. 20057243 10.12927/hcq.2009.21091

[16] Gröne, O and Garcia-Barbero, M. Integrated care: a position paper of the WHO European Office for Integrated Health Care Services. Int J Integr Care, 2001; 1(June): e21. 16896400 PMC1525335

[17] PATH. PATH’s framework for health services integration Seattle, WA; 2011.

[18] World Health Organization. Framework on integrated, people-centred health services – Report. World Heal Assem [Internet], 2016; (A69/39): 1–12. Available from: http://apps.who.int/gb/ebwha/pdf_files/WHA69/A69_39-en.pdf?ua=1.

[19] Siegel, S, Alderwick, H, Vuik, S, Ham, C and Patel, H. Healthy populations: Designing strategies to improve population health; 2016 WISH – World Innovation Summit for Health, 2016.

[20] Koch, U, Stout, S, Landon, BE and Phillips, RS. From healthcare to health: A proposed pathway to population health. Healthcare. 2016; 4(4): 291–7. DOI: 10.1016/j.hjdsi.2016.06.007 27693259

[21] Public Health Agency of Canada. Population Health Approach: The Organizing Framework [Internet]. Canadian Best Practice Portal; 2013 Available from: http://cbpp-pcpe.phac-aspc.gc.ca/population-health-approach-organizing-framework/.

[22] The Federal Provincial and Territorial Advisory Committee on Population Health (ACPH). Strategies for population health--investing in the health of Canadians. Probe [Internet], 1994; 30(3). Available from: http://www.ncbi.nlm.nih.gov/pubmed/9611430.

[23] Ham, C and Alderwick, H. Place-based systems of care, a way forward for the NHS in England London, UK: The King’s Fund; 2015.

[24] Vaida, B. For Super-Utilizers, Integrated Care Offers A New Path. Health Aff [Internet], 2017; 36(3): 394–7. Available from: http://content.healthaffairs.org/lookup/doi/10.1377/hlthaff.2017.0112 DOI: 10.1377/hlthaff.2017.0112 28264939

[25] Pines, J, Selevan, J, McStay, F, George, M and McClellan, M. Kaiser Permanente – California: A Model for Integrated Care for the Ill and Injured; 2015.

[26] Hildebrandt, H, Pimperl, A, Schulte, T, Hermann, C, Riedel, H, Schubert, I, et al. [Pursuing the triple aim: evaluation of the integrated care system Gesundes Kinzigtal: Population health, patient experience and cost-effectiveness]. Triple Aim – Eval der Integr Versorgung Gesundes Kinzigtal – Gesundheitszustand, Versorgungserleb und Wirtschaftlichkeit [Internet], 2015; 58(4–5): 383–92. Available from: http://ovidsp.ovid.com/ovidweb.cgi?T=JS&PAGE=reference&D=prem&NEWS=N&AN=25652116 DOI: 10.1007/s00103-015-2120-y 25652116

[27] Arksey, H and O’Malley, L.Scoping studies: towards a methodological framework. Int J Soc Res Methodol [Internet], 2005 2; 8(1): 19–32. Available from: http://www.tandfonline.com/doi/abs/10.1080/1364557032000119616 DOI: 10.1080/1364557032000119616

[28] Kodner, DL and Spreeuwenberg, C. Integrated care: meaning, logic, applications, and implications – a discussion paper. Int J Integr Care [Internet], 2002; 2: e12 Available from: http://www.ncbi.nlm.nih.gov/pmc/articles/PMC1480401/ DOI: 10.5334/ijic.67 16896389 PMC1480401

[29] Public Health Agency of Canada. What Determines Health? [Internet]. Population Health; 2011 [cited 2016 6 24]. Available from: http://www.phac-aspc.gc.ca/ph-sp/determinants/index-eng.php.

[30] Leutz, W. Five Laws for Integrating Medical and Social Services: Lessons from the United States and the United Kingdom. Milbank Q [Internet], 1999; 77(1): 77–110. Available from: http://ovidsp.ovid.com/ovidweb.cgi?T=JS&PAGE=reference&D=ovftc&NEWS=N&AN=00005716-199903000-00004 DOI: 10.1111/1468-0009.00125 10197028 PMC2751110

[31] Fulop, N, Mowlem, A and Edwards, N. Building integrated care: Lessons from the UK and Elsewhere. NHS Confed London, 2005; 3–15.

[32] Billings, J and de Weger, E. Contracting for integrated health and social care: A critical review of four models. J Integr Care [Internet], 2015; 23(3): 153–75. Available from: http://0-www.emeraldinsight.com.lispac.lsbu.ac.uk/doi/10.1108/JICA-03-2015-0015 DOI: 10.1108/JICA-03-2015-0015

[33] Béland, F, Bergman, H, Lebel, P, Clarfield, AM, Tousignant, P, Contandriopoulos, A-P, et al. A system of integrated care for older persons with disabilities in Canada: Results from a randomized controlled trial. J Gerontol A Biol Sci Med Sci, 2006; 61(4): 367–73. DOI: 10.1093/gerona/61.4.367 16611703

[34] Wynia, K, Kremer, B, Spoorenberg, S, Uittenbroek, R and Reijneveld, S. Embrace: a population based Integrated Elderly Care model showed improved results and saves costs. Eur J Public Health, 2014; 24(1). DOI: 10.1093/eurpub/cku164.021

[35] Gray, BH, Winblad, U and Sarnak, DO. Sweden’s Esther Model: Improving Care for Elderly Patients with Complex Needs. Commonw Fund, 2016; 29(9).

[36] Collins, B. Intentional whole health system redesign: Southcentral Foundation’s “Nuka” system of care King’s Fund [Internet], 2015; 11 Available from: http://www.kingsfund.org.uk/sites/files/kf/field/field_publication_file/intentional-whole-health-system-redesign-Kings-Fund-November-2015.pdf?utm_source=The King’s Fund newsletters&utm_medium=email&utm_campaign=6430636_HMP 2015-10-20&dm_i=21A8,3TTWS,FLX.

[37] Hildebrandt, H, Hermann, C, Knittel, R, Richter-Reichhelm, M, Siegel, A, Witzenrath, W, et al. Gesundes Kinzigtal Integrated Care: Improving population health by a shared health gain approach and a shared savings contract. Int J Integr Care [Internet], 2010; 10(6): e046 Available from: http://ovidsp.ovid.com/ovidweb.cgi?T=JS&PAGE=reference&D=prem&NEWS=N&AN=20689772 DOI: 10.5334/ijic.539 20689772 PMC2914874

[38] Henry, SL, Shen, E, Ahuja, A, Gould, MK and Kanter, MH. The online personal action plan: A tool to transform patient-enabled preventive and chronic care. Am J Prev Med [Internet], 2016; 51(1): 71–7. DOI: 10.1016/j.amepre.2015.11.014 26826751

[39] Daniilidou, NV, Economou, C, Zavras, D, Kyriopoulos, J and Georgoussi, E. Health and social care in aging population: An integrated care institution for the elderly in Greece. Int J Integr Care [Internet], 2003; 3(10): e04 Available from: http://www.pubmedcentral.nih.gov/articlerender.fcgi?artid=1483938&tool=pmcentrez&rendertype=abstract DOI: 10.5334/ijic.92 16900264 PMC1483938

[40] Tuso, P. Physician update: Total health. Perm J, 2014; 18(2): 58–63. DOI: 10.7812/TPP/13-120 PMC4022559 24694316

[41] Storey-Kuyl, M, Bekemeier, B and Conley, E. Focusing “upstream” to Address Maternal and Child Health Inequities: Two Local Health Departments in Washington State Make the Transition. Matern Child Heal J [Internet], 2015 11; 19(11): 2329–2335 7p. Available from: http://search.ebscohost.com/login.aspx?direct=true&db=cin20&AN=110164581&site=ehost-live DOI: 10.1007/s10995-015-1756-4 26082170

[42] Porter Kellog, MM. Kaiser Permanente: An Integrated Health Care Experience. Rev Innovación Sanit y Atención Integr, 2008; 1(1): 1–8.

[43] Kanter, MH, Lindsay, G, Bellows, J and Chase, A. Complete care at Kaiser permanente: Transforming chronic and preventive care. Jt Comm J Qual Patient Saf, 2013; 39(11): 484–94. DOI: 10.1016/S1553-7250(13)39064-3 24294676

[44] Hildebrandt, H, Schulte, T and Stunder, B. Triple Aim in Kinzigtal, Germany Improving population health, integrating health care and reducing costs of care – lessons for the UK? J Integr Care [Internet], 2012; 20(4): 205–22. Available from: http://www.emeraldinsight.com/doi/abs/10.1108/14769011211255249 DOI: 10.1108/14769011211255249

[45] Bullen, C, Kearns, RA, Clinton, J, Laing, P, Mahoney, F and McDuff, I. Bringing health home: Householder and provider perspectives on the healthy housing programme in Auckland, New Zealand. Soc Sci Med, 2008; 66(5): 1185–96. DOI: 10.1016/j.socscimed.2007.11.038 18191008

[46] Amelung, VE, Reichert, A, Urbanski, D, Matejevic, L, O’riordan, E, Blatt, E, et al. Integrating Health and Social Care A global perspective of experience, best practices and the way forward; INAV – Institute for Applied Health Services Research 2014.

[47] Baker, GR, MacIntosh-Murray, A, Porcellato, C, Dionne, L, Stelmacovich, K and Born, K. Jönköping County Council. High Perform Healthc Syst [Internet], 2008; 121–44. Available from: http://www.longwoods.com/product/download/code/20155.

[48] Jackson, G, Thornley, S, Woolston, J, Papa, D, Bernacchi, A and Moore, T. Reduced acute hospitalisation with the healthy housing programme. J Epidemiol Community Heal [Internet], 2011; 65(7): 588–93. Available from: http://jech.bmj.com/cgi/doi/10.1136/jech.2009.107441 DOI: 10.1136/jech.2009.107441 21282140

[49] Watson, I. Liverpool Healthy Homes Programme Liverpool (England): Liverpool City Council; 2011.

[50] Harris, M, Greaves, F, Patterson, S, Jones, J, Pappas, Y, Majeed, A, et al. The North West London Integrated Care Pilot: Innovative strategies to improve care coordination for older adults and people with diabetes. J Ambul Care Manage, 2012; 35(3): 216–25. DOI: 10.1097/JAC.0b013e31824d15c7 22668611

[51] Grilz-wolf, M. Providing integrated health and social care for older persons in Austria. PROCARE – Providing integrated health and social care for older persons; 2003.

[52] Van Vliet, K and Oudenampsen, D. Integrated care in the Netherlands Utrecht: Verwey-Jonker Instituut; 2004.

[53] Siegel, A and Stossel, U. Evaluation of the Gesundes Kinzigtal Integrated Care: Results until now [Internet]. Public Health Forum, Siegel, A, Lehrbereich Allgemeinmedizin, Universitatsklinikum Freiburg im Breisgau, Elsasser Str. 2m, Haus 1A, DG 79110 Freiburg, Germany. E-mail: achim.siegel@uniklinik-freiburg.de: Urban und Fischer Verlag Jena (P.O. Box 100537, Jena 07705, Germany), 2013; 13 (Evaluation der Integrierten Versorgung Gesundes Kinzigtal: Bisherige Ergebnisse; vol. 21). Available from: http://ovidsp.ovid.com/ovidweb.cgi?T=JS&PAGE=reference&D=emed11&NEWS=N&AN=2013192226 DOI: 10.5334/ijic.432

[54] Spoorenberg, SLW, Uittenbroek, RJ, Kremer, HPH, Reijneveld, SA and Wynia, K. Advantages of person-centered and integrated care service: results of Mixed Method Research on Embrace. Int J Integr Care [Internet], 2016; 16(6): 226. Available from: https://www.ijic.org/article/10.5334/ijic.2774/ DOI: 10.5334/ijic.2774

[55] Jones, M. Nuka-style models of primary healthcare. Pract Manag, 2017; 27(4): 26–9. DOI: 10.12968/prma.2017.27.4.26

[56] Staines, A, Thor, J and Robert, G. Sustaining improvement? the 20-year jönköping quality improvement program revisited. Qual Manag Health Care, 2015; 24(1): 21–37. DOI: 10.1097/QMH.0000000000000048 25539488

[57] Blewett, LA and Owen, RA. Accountable care for the poor and underserved: Minnesota’s Hennepin Health model. Am J Public Health [Internet], 2015; 105(4): 622–4. Available from: http://ajph.aphapublications.org/doi/abs/10.2105/AJPH.2014.302432 DOI: 10.2105/AJPH.2014.302432 25713963 PMC4358174

[58] Bellows, J, Young, S and Chase, A. Person-focused care at kaiser permanente. Perm J [Internet], 2014; 18(1): 90–1. Available from: http://www.pubmedcentral.nih.gov/articlerender.fcgi?artid=3951036&tool=pmcentrez&rendertype=abstract DOI: 10.7812/TPP/13-165 24626077 PMC3951036

[59] Johnson, N. Liverpool Healthy Homes programme: delivering NICE Guideline NG6 “Excess winter deaths and morbidity and the health risks associated with cold homes” [Internet]. NICE – National Institute for Health and Care Excellence; 2016 Available from: https://www.nice.org.uk/sharedlearning/liverpool-healthy-homes-programme-7-years-of-pre-empting-nice-guidline-ng6-excess-winter-deaths-and-morbidity-and-the-health-risks-associated-with-cold-homes#results.

[60] Hildebrandt, H, Schulte, T and Stunder, B. Triple Aim in Kinzigtal, Germany Improving population health, integrating health care and reducing costs of care – lessons for the UK? J Integr Care [Internet], 2012; 20(4): 205–22. Available from: http://search.ebscohost.com/login.aspx?direct=true&db=c8h&AN=2011695269&site=ehost-live DOI: 10.1108/14769011211255249

[61] Bodenheimer, T, Bojestig, M and Henriks, G. Making systemwide improvements in health care: Lessons from Jonkoping County, Sweden. Qual Manag Health Care, 2007; 16(1): 10–5. DOI: 10.1097/00019514-200701000-00003 17235247

[62] Spoorenberg, SLW, Uittenbroek, RJ, Middel, B, Kremer, BPH, Reijneveld, SA and Wynia, K. Embrace, a model for integrated elderly care: Study protocol of a randomized controlled trial on the effectiveness regarding patient outcomes, service use, costs, and quality of care. BMC Geriatr, 2013; 13(1): 62 DOI: 10.1186/1471-2318-13-62 23782932 PMC3702391

[63] Sandberg, SF, Erikson, C, Owen, R, Vickery, KD, Shimotsu, ST, Linzer, M, et al. Hennepin health: A safety-net accountable care organization for the expanded medicaid population. Health Aff, 2014; 33(11): 1975–84. DOI: 10.1377/hlthaff.2014.0648 25367993

[64] Halfon, N, Larson, K and Russ, S. Why Social Determinants. Healthc Q, 2010; 14(Special Issue October): 8–20. 20959743 10.12927/hcq.2010.21979

[65] Powell Davies, G, Williams, AM, Larsen, K, Perkins, D, Roland, M and Harris, MF. Coordinating primary health care: An analysis of the outcomes of a systematic review. The Medical Journal of Australia, 2008; 188. 10.5694/j.1326-5377.2008.tb01748.x 18429740

[66] Lewis, R, Rosen, R, Goodwin, N and Dixon, J. Where next for integrated care organisations in the English NHS? [Internet]. The Nuffield Trust, 2010; 1–38. Available from: http://www.nuffieldtrust.org.uk/sites/files/nuffield/publication/where_next_for_integrated_care_organisations_in_the_english_nhs_230310.pdf.

[67] Berwick, DM, Nolan, TW and Whittington, J. The triple aim: Care, health, and cost. Health Aff, 2008; 27(3): 759–69. DOI: 10.1377/hlthaff.27.3.759 18474969

[68] Aase, K, Waring, J and Schibevaag, L. Researching quality in care transitions: International perspectives Cham: Springer International Publishing AG; 2017 DOI: 10.1007/978-3-319-62346-7

[69] McCarthy, D, Mueller, K and Wrenn, J. Case Study Kaiser Permanente: Bridging the Quality Divide with Integrated Practice, Group Accountability, and Health Information Technology. Commonw Fund [Internet], 2009; 17(June): 1–45. Available from: http://www.commonwealthfund.org/~/media/Files/Publications/Case Study/2009/Jun/1278_McCarthy_Kaiser_case_study_624_update.pdf.

[70] Tierney, S and Odden, T. Nuka System of Care: Using Data for improvement. In: 16th Annual Summit [Internet]; 2015 Available from: http://www.kingsfund.org.uk/publications/population-health-systems/nuka-system-care-alaska http://www.kingsfund.org.uk/publications/population-health-systems http://www.kingsfund.org.uk/blog/2014/03/innovation-alaska-walking-with-communities-achieve.

[71] Busse, R and Stahl, J. Integrated care experiences and outcomes in Germany, the Netherlands, and England. Health Aff, 2014; 33(9): 1549–58. DOI: 10.1377/hlthaff.2014.0419 25201659

[72] Gottlieb, K. The Nuka System of Care: Improving health through ownership and relationships. Int J Circumpolar Health, 2013; 72(Suppl 1). DOI: 10.3402/ijch.v72i0.21118 PMC3752290 23984269

[73] Etches, V, Frank, J, Di Ruggiero, E and Manuel, D. Measuring population health: a review of indicators. Annu Rev Public Health [Internet], 2006; 27: 29–55. Available from: http://www.ncbi.nlm.nih.gov/pubmed/16533108 DOI: 10.1146/annurev.publhealth.27.021405.102141 16533108

[74] Local Government Association. Healthy homes, healthy lives London, UK; 2014.

[75] Barr, VJ, Robinson, S, Marin-Link, B, Underhill, L, Dotts, A, Ravensdale, D, et al. The expanded Chronic Care Model: an integration of concepts and strategies from population health promotion and the Chronic Care Model. Hosp Q, 2003; 7(November 2003): 73–82. DOI: 10.12927/hcq.2003.16763 14674182

[76] Norris, T. Total Health: Public Health and Health Care. In: A Healthier America 2013: Strategies To Move From Sick Care To Health Care In The Next Four Years. Trust for America’s Health, 2013; 25–7.

[77] Andermann, A. Taking action on the social determinants of health in clinical practice: a framework for health professionals. Can Med Assoc J, 2016; 1–10. DOI: 10.1503/cmaj.160177 PMC5135524 27503870

[78] Gozzard, D, Willson, A, Henriks, G, Asaad, K, Crosby, T, Harrison, J, et al. Quality, Development and Leadership-Lessons to learn from Jönköping Cardiff, UK, 2011; 1000(4): 1–21.

[79] Hostetter, M, Klein, S and Mccarthy, D. Hennepin Health: A Care Delivery Paradigm for New Medicaid Beneficiaries, 2016; 33 The Commonwealth Fund.

[80] Sissouras, A, Ketsetzopoulou, M, Bouzas, N, Fagadaki, E, Papaliou, O and Fakoura, A. Providing integrated health and social care for older persons in Greece National Centre for Social Research; 2003.

[81] Johri, M, Beland, F and Bergman, H. International experiments in integrated care for the elderly: A synthesis of the evidence. Int J Geriatr Psychiatry, 2003; 18(3): 222–35. DOI: 10.1002/gps.819 12642892

[82] Wallace, P, Michener, L and Wellik, M. Primary Care and Public Health: Exploring Integration to Improve Population Health – Institute of Medicine [Internet]; 2012 Available from: http://www.iom.edu/Reports/2012/Primary-Care-and-Public-Health.aspx. 24851288

[83] Levine, JF, Herbert, B, Mathews, J, Serra, A and Rutledge, V. Use of the Triple Aim to improve population health. N C Med J [Internet], 2011; 72(3): 201–4. Available from: http://www.ncbi.nlm.nih.gov/pubmed/21901915. 21901915

